# Surgery and Radiosurgery for Acromegaly: A Review of Indications, Operative Techniques, Outcomes, and Complications

**DOI:** 10.1155/2012/386401

**Published:** 2012-03-01

**Authors:** Yvette Marquez, Alexander Tuchman, Gabriel Zada

**Affiliations:** Department of Neurosurgery, Keck School of Medicine, USC Medical Center, University of Southern California, 1200 North State Street, Suite 3300, Los Angeles, CA 90033, USA

## Abstract

Among multimodality treatments for acromegaly, the goals of surgical intervention are to balance maximal tumor resection while preserving normal pituitary function and maintaining patient safety. The resection of growth hormone-(GH-) secreting pituitary adenomas in the hands of experienced surgeons results in hormonal remission in 50–70% of patients. Acromegalic patients often have medical comorbidities and anatomical variations complicating anesthesia and surgical management. Despite these challenges, complications such as CSF leak or new hypopituitarism following surgery remain uncommon. Over the past decade, endoscopic approaches to pituitary tumors have improved visualization and facilitated identification of additional tumor using angled telescopes. Patients with persistent acromegaly following surgery require continued medical and/or radiation-based interventions. The adjunctive use of stereotactic radiosurgery offers hormonal remission in 40–50% of patients. In this article, the current preoperative evaluation, indications for surgery, surgical approaches, role of radiosurgery, complications, and remission criteria following operative resection of GH adenomas are reviewed.

## 1. Introduction

Acromegaly is a life-threatening disorder resulting from excessive growth hormone (GH) secretion, occurring almost exclusively secondary to pituitary adenomas. Although other causes rarely exist, pituitary adenomas are the most common culprit of this systemic condition that frequently leads to hypertension, cardiac disease, diabetes, pulmonary disorders, and associated malignancies [1]. The annual incidence of acromegaly has been calculated to be 3-4 cases per 1 million people, with a prevalence of 40–60 per million people [2, 3]. Because of its insidious development and the slow progression of clinical manifestations, acromegaly frequently remains undiagnosed for many years. Moreover, on account of the common delay in diagnosing GH adenomas, the majority are macroadenomas (>10 mm) at the time of diagnosis.

Prompt treatment should be initiated for most patients with acromegaly, as the mortality rate for uncontrolled disease is 2–4 times higher than that of the general population [4–6]. Cardiovascular disease comprises 60% of acromegaly-related deaths, with the remaining 25% and 15% accounted for by respiratory disease, and malignancies, respectively [7]. Specifically, lung and bowel cancers account for almost 70% of all neoplasms, and cancer mortality is 4.6-fold-higher in acromegalic men than in the normal male population. The overall treatment goal in patients with acromegaly is to achieve normalization of age, and sex-specific serum IGF-1 levels, as this may reverse the increased mortality rate associated with this disease. One of the most effective and rapid ways of accomplishing this goal, and often the preferred initial treatment, is via surgical intervention. When possible, total resection of the tumor mass and subsequent normalization of serum IGF-1 levels often leads to reversal of associated symptoms (including headache and visual impairment), alleviation of comorbidities such as hypertension and endocrine abnormalities, and changes in some of the somatic features of the disease [8, 9].

While surgery is at the forefront of the treatment paradigm, a major role for medical management still exists in patients with acromegaly. Medications for acromegaly are typically indicated for patients that are not surgical candidates or do not wish to have surgery, following subtotal tumor resection, and for patients with persistent elevations in GH and IGF-1 levels that are awaiting the effectiveness of radiation-based treatments. Medical management may also be indicated in patients with large GH-secreting macroadenomas that are not likely to be cured by surgery, in which a decision to forego an attempt at surgical debulking is made. First-line medical therapy for acromegaly frequently includes somatostatin analogs, such as octreotide or lanreotide. Pegvisomant, the newest medical treatment available for GH adenomas, blocks the hormonal action of GH at the level of GH receptors in the liver and has been shown to normalize IGF-1 in 97% of patients after one year of administration [3]. Despite their poor efficacy and side effect profiles, dopamine agonists medications, such as bromocriptine and cabergoline, have been shown to normalize IGF-1 in a minority of patients [3, 10, 11].

## 2. Preoperative Evaluation

The diagnosis of acromegaly is based on the demonstration of high circulating levels of GH and IGF-I, with the latter being the current gold standard for diagnosing the disease. In order to exclude acromegaly as a diagnosis, a single random GH level lower than 0.4 *μ*g/L and an IGF-I value in the age- and sex-matched normal range must be met. In uncertain cases (i.e., when these two criteria are not met for exclusion), a 75 g oral glucose test (OGTT) should be conducted. The lack of serum GH suppressibility below 1 *μ*g/L (0.3 *μ*g/L according to recent reports) within two hours after an oral glucose load will confirm the diagnosis [12].

In greater than 90% of patients, acromegaly is caused by a pituitary adenoma [10, 13, 14]. Other causes of acromegaly include tumors of the pancreas, lungs, and adrenal glands. Magnetic resonance imaging (MRI) of the sellar region, with and without gadolinium contrast administration, is the gold standard imaging study for diagnosis and preoperative assessment of these pituitary adenomas. Specifically, information regarding tumor size and location can have tremendous value during and after surgical intervention. Moreover, intracavernous extension (ICE), which often hinders the complete removal of pituitary adenomas, should be assessed. GH adenomas have a demonstrated propensity to invade the infrasellar region, often growing through the sellar dura and bony floor into the sphenoid sinus [14]. If the tumor extends into the suprasellar region, an ophthalmological examination to evaluate for visual field defects should be acquired prior to treatment. While the utility of MRI in diagnosing the primary cause of acromegaly cannot be overstated, it should be noted that the ability to predict surgical outcomes on the basis of preoperative pituitary MRI is still being investigated. Nevertheless, recent studies have shown that preoperative MRI is very useful for the prediction of certain surgical results. For example, cure is much less likely in patients harboring tumors greater than 15 mm in diameter, those with suprasellar extension above the optic chiasm, and in the setting of extensive intracavernous extension [13].  Table 1 provides other preoperative predictors that have been proposed. It should be noted that because patients with acromegaly usually have atypical vascular and bony anatomy, additional imaging (i.e., CT or CT angiography) may be necessary to plan the proper approach, assess the degree of bony disease, and avoid vascular injuries [14].

## 3. Indications for Surgery

Surgery is the first line treatment for most patients with acromegaly. Indications for surgery include active acromegaly with an appropriate surgical target, visual loss, and other forms of mass effect, pituitary tumor apoplexy, and refractory or progressive disease despite treatment attempts using other therapies. Some contraindications to surgery include advanced age, general debility, or other commonly occurring medical comorbidities (i.e., cardiovascular or pulmonary disease) [9]. Preoperative evaluation in patients with acromegaly should also include a detailed assessment of clinical features (i.e., enlargement of facial features, cutaneous changes, arthropathies, changes in cardiovascular system, and signs and symptoms of hypopituitarism). Airway evaluation by an anesthesiologist is often required, as many acromegalic patients have significant tongue and airway edema that may require specialized intubation.

## 4. Surgical Approach

Surgical approaches in patients with acromegaly have evolved dramatically since Drs. Caton and Paul first attempted to resect a pituitary tumor in 1893 [9]. A major driving force behind the evolution of these techniques has been improvement in surgical optics and instrumentation, which have fueled the trend towards progressive minimalization. Generally speaking, access to the pituitary region involves either an open craniotomy or a transsphenoidal approach. The transphenoidal approach can be completed in two distinct fashions: via a microscope or with the assistance of an endoscope.

## 5. Transsphenoidal Surgery

Transsphenoidal surgery, specifically transsphenoidal microsurgical adenectomy, has become the primary surgical approach for treating pituitary adenomas over the past several decades [9, 15, 16]. The other major option for large tumors with extensive suprasellar or lateral extension is an open craniotomy (see below). Certain factors that must be taken into account when deciding on a particular surgical approach include the anatomy of the sinuses and pituitary region (i.e., size and morphology of the sella, pneumatization of air sinuses, etc.), the surgeon's experience, and extent of tumor burden [9, 17]. With regard to the transsphenoidal approach, an assessment of the pattern of extrasellar extension on preoperative MR images is essential prior to resection of pituitary adenomas, in order to delineate which regions pose the greatest limitation for tumor resection and are likely to retain residual tumor [14, 18].

## 6. Endoscopic Transsphenoidal Surgery

Minimally invasive techniques have been at the forefront of approaches to the pituitary gland. In recent years, the use of endoscopes has become commonplace during transsphenoidal pituitary adenoma resection. Pure endoscopic approaches offer the benefits of improved illumination and magnification, panoramic visualization, angled endoscopes to “look around corners” for residual tumor, and obviate the need for nasal retractors or nasal packing in most cases. Furthermore, the use of endoscopes offers a wider range of targets along the arc of the skull base, from the frontal sinuses anteriorly and superiorly to the craniocervical junction posteriorly and inferiorly.

## 7. Craniotomy

For GH adenomas that have suprasellar extension or are mainly localized outside of the sella, transcranial approaches are often necessary for improved visualization of the pituitary tumor. With a frontotemporal craniotomy, once the frontal lobes have been carefully retracted, the surgeon can directly see the optic nerves or chiasm and any extension of the pituitary tumor into the anterior fossa, middle fossa, or suprasellar region [9, 19]. A supraorbital “eyebrow” keyhole craniotomy may also be utilized to approach the suprasellar region and optic apparatus and can also be used in conjunction with the endoscope. Other indications for transcranial approaches have traditionally included a small sella turcica with a large extrasellar component, unfavorable or nonpneumatized parasellar nasal sinuses, coexisting ICA or ACA aneurysm or other vascular anomalies, or firm or fibrous pituitary adenomas (identified during a previous procedure). It should be noted, however, that as technical advances are made in the endoscopic transsphenoidal approach, the limitations and contraindications associated with transsphenoidal surgery continue to diminish [19].

## 8. Surgical Results

### 8.1. Surgical Outcomes

Although transsphenoidal craniotomy (TSC) is considered the gold standard intervention for the treatment of acromegaly, it is imperative that the goal of treatment—the restoration of a normal GH secretion profile and normalization of serum IGF-1—not be overlooked [13]. In the majority of cases, surgery provides long-term remission in 70–88% of patients with microadenomas and 50–61% of patients with macroadenomas. The ability to achieve hormonal remission following TSC is often limited by tumor size (i.e., macroadenomas ≥ 10 mm diameter), the degree of extrasellar extension (particularly lateral into the cavernous sinuses), and high preoperative serum GH concentrations (≥45 ng/mL) [7, 16, 20]. Laws et al. characterized microscopic invasiveness of the tumor as a factor that is also associated with outcomes following transsphenoidal surgery for acromegaly. In their analysis, hormonal recurrence after 10-year follow-up occurred in 8–10% of patients and was more likely in tumors that invaded the parasellar dura or bone [15].

#### 8.1.1. Risks and Complications Associated with Surgical Approaches for Acromegaly

Aside from the perioperative medical risks inherent to patients with acromegaly, many risks associated with transsphenoidal operations for acromegaly may be heightened on account of the anatomical deviations occurring frequently in this population. Common factors that may affect the intraoperative plan include airway/laryngeal edema, cardiopulmonary dysfunction, and venous congestion. During the approach itself, the initial visualization can be limited by mucosal edema/redundant tissue, hypertrophic nasal turbinates, and thickened nasal bones (i.e., the sphenoid rostrum and vomer). In addition, thickened sphenoid septa and mucosa may lead to restricted workspace within the sinus and excessive mucosal bleeding. Lastly, patients with longstanding acromegaly often have enlargement and tortuosity of the internal carotid arteries and occasionally have aneurismal formations [9, 14, 15]. Therefore, it is important to assess the course of the ICA within the parasellar nasal and cavernous sinuses prior to selecting a surgical approach.

Complications that are associated with transcranial approaches for GH-denomas include hemorrhage, infection, or stroke secondary to brain retraction/ischemia. In acromegalic patients, the frontal sinuses are often unusually large, and violation of a frontal sinus during a cranial approach can lead to a cerebrospinal fluid leak and subsequent meningitis if not properly identified and repaired during surgery. Close scrutiny of preoperative imaging studies and implementation of various intraoperative maneuvers may optimize the safety profiles associated with any surgical procedure for GH adenomas [9]. 

Despite the prevalence of medical comorbidities in this population, overall morbidity rates associated with the surgical management of acromegaly remain extremely low, and mortality has been reported in less than 0.5% of patients treated in high-volume centers [3, 15, 19, 21]. The most common postoperative patient complaints are usually due to sinonasal causes (approx. 60% in one recent study) [22], with nasal congestion (42%), alteration in taste or smell (30%), sinusitis (30%), and epistaxis (6.7%) occurring most frequently [22]. Cerebrospinal fluid leakage is the next most common complication and has been reported to occur in 1–5% of cases [3, 10]. Transient endocrine disturbances such as diabetes insipidus (DI) have been noted in approximately 3% of cases. Moreover, DI develops more commonly following transcranial approaches than transsphenoidal approaches, typically as a result of manipulation of the pituitary stalk or hypothalamus. Permanent DI is rare, developing in 1-2% of patients following surgery. The syndrome of inappropriate antidiuretic hormone hypersecretion (SIADH) resulting in hyponatremia has been shown to occur in 8% of patients and typically occurs in a delayed fashion approximately one week following the operation [13, 23].

Although visual loss and hypopituitarism may result from tumors causing acromegaly, they can also result from iatrogenic injury during surgical tumor resection. Both hypopituitarism and visual loss occur much more frequently in patients with macroadenomas [6, 24]. The evaluation of pituitary function and visual fields are important in the preoperative assessment of patients with acromegaly and may serve as baseline studies from which to identify improvements or complications occurring following surgery. Visual loss has been reported in 5% of patients following transsphenoidal surgery, while new hypopituitarism may develop in 10–20% of patients [6, 25]. Vascular injury, specifically to the internal carotid arteries, is one of the most serious complications of surgical intervention for acromegaly and may result in a pseudoaneurysm, stroke, or death. The incidence of carotid artery injury during transsphenoidal surgery, however, is less than 0.5%. Post-operative hemorrhage into the tumor resection cavity can lead to worsening pressure on the optic nerves or chiasm and possibly visual loss, is associated with subtotal tumor resection, and mandates urgent surgical evacuation.

## 9. Multimodal Therapy and the Role of Radiosurgery

Multiple radiation-based treatment strategies have been described for residual or recurrent pituitary adenomas. Nearly 75% of GH producing tumors are macroadenomas at the time of diagnosis, and often invade the surrounding dura and bone, making complete surgical resection nearly impossible without compromising patient safety [26]. Following microsurgical resection, 10–50% of patients will have persistent elevations in GH and/or IGF-1 levels [9, 27, 28]. Furthermore, the recurrence rate following initial hormonal remission is 8–20% in patients with acromegaly [15, 27, 29]. In GH-producing tumors following subtotal surgical resection or disease recurrence, radiation treatment, and medical therapy can be used either separately, or in conjunction, as adjunctive modalities to achieve endocrine remission and control tumor growth. The benefits of radiation-based treatments, such as external beam therapy and radiosurgery, must always be weighed against inherent risks such as new hypopituitarism, visual loss, and cranial nerve deficits.

Fractionated external radiotherapy has been described in the treatment of somatotrophic adenomas with varied success rates (5–74% hormonal remission), a long latency to functional response (5–15 years), and high rates of pituitary dysfunction (50–80%) [28, 38, 39]. The relatively high rates of adverse effects associated with radiotherapy have limited its use in recent years, with treatment practices shifting towards radiosurgery in the majority of major treatment centers. Stereotactic radiosurgery was first described by Lars Lesksel in 1951 and has been shown to be effective in the treatment of a variety of intracranial tumors [40]. Radiosurgery uses multiple low energy radiation beams designed to converge on a stereotactic target, resulting in high-dose radiation specific to the target with a sharp falloff in exposure outside the target. The radiation may be delivered as photons in Gamma Knife Surgery (GKS) and linear particle accelerator-(LINAC-) based radiosurgery (e.g., CyberKnife), or as charged particles in the case of proton-beam radiosurgery. Although treatment of acromegaly has been described with LINAC and proton-beam radiosurgery, few studies with small numbers of patients have limited significant evaluation of their effectiveness as compared to GKS, which many studies have evaluated [10, 26–28, 30, 32, 34, 35, 41–47]. GKS consists of a frame-based single session treatment, with mean marginal radiation doses for growth hormone adenomas typically ranging between 14–34 Gy to the 50% isodose line [48].

GKS has been found to be effective in the treatment of acromegaly in multiple large cohorts [26, 27, 30, 32, 35–37, 43, 46, 47, 49]. Among published case series of GH adenomas treated with radiosurgery, tumor control (stable or decreased tumor volume) was achieved in 97% of patients [48]. Radiosurgery has been less successful in achieving endocrinological remission, defined as normalization of age- and sex-appropriate IGF-1 and GH levels, with hormonal remission rates among larger case series being 17–96% [26, 27, 30–32, 34–37, 43, 46, 49]. This large variation among studies reporting results of radiosurgery for acromegaly may be attributed to differences in hormonal remission criteria (threshold of GH or IGF-1 levels), treatment plans (radiation dose), and length of followup time. According to a meta-analysis of 970 patients in a recent review, endocrine remission was achieved in 48–53% of patients after radiosurgery, with an overall disease control rate (with or without ongoing medical therapy) of 73% [50]. Jagannathan et al. reported a median time to remission of 29.8 months following GKS [26, 51]. Reported factors associated with postradiosurgical endocrine remission include higher prescribed radiation dose, lower preradiosurgery GH and IGF-1 levels, and holding hormone suppressive medications prior to radiosurgery [27, 30, 37, 43, 46].

It has been reported that some medications used to treat acromegaly, especially somatostatin-analogos, may make tumors less susceptible to radiosurgery by altering tumor metabolism dynamics and the cell cycle [26, 46, 51, 52]. Pollock et al. described a series of 31 patients treated with octreotide at the time of radiosurgery, in which the time to remission was significantly longer than in patients not receiving octreotide at the time of treatment [46]. Pollock et al. reported that holding growth hormone suppressive medications at the time of GKS correlated with biochemical remission [46]. This has led some groups to recommend holding somatostatin-analogs for at least a month prior to radiosurgery, based on research showing long acting somatostatin-analogs such as Sandostatin LAR may exert a suppressive effect on GH release for 8–12 weeks [46, 52, 53]. The optimal timing for withholding suppressive medications is still under investigation, but most groups recommending this practice suggest a time of 4–8 weeks prior to GKS. 

Although, in general, radiosurgical treatments offer safe treatment profiles with few side effects, complications associated with treatment do occur and must be considered seriously prior to treatment. Common complications of stereotactic radiosurgery include pituitary hormone deficiency, visual disturbances, and cranial nerve deficits. New pituitary hormone deficiency is the most frequently reported complication of radiosurgery for parasellar tumors, affecting 2–47% of patients [26, 27, 30–32, 34–37, 43, 46, 47]. Pituitary hormone deficiency tends to occur in a delayed fashion and is usually first observed at 1.5–5 years after radiosurgery [26, 52]. Thus, in studies with longer followup, the rate of pituitary dysfunction tends to increase. Larger tumor volume, specifically greater than 4 cm^3^, has been correlated with a higher incidence of hormone deficiency after GKS [52, 54]. This is likely related to the larger treatment volume required in these cases. Therefore, a common paradigm for the treatment of pituitary adenomas consists of tumor debulking prior to GKS [52]. In general, visual complications occur rarely following GKS, with the majority of series having no cases of visual loss reported. In those cohorts that did contain visual complications, the reported rate ranged between 1–11% [26, 32, 34, 35, 37]. Strategies to minimize visual loss associated with GKS include limiting its use to lesions at least 3 mm from the optic nerves or chiasm [45]. For those tumors <3 mm from optic structures, multisession frameless radiosurgery (i.e., Cyberknife) may be a safe alternative [55]. Cranial nerve deficits were reported in only three of 45 case series, according to a recent review [48]. Other rarely reported complications of radiosurgery include headache, trigeminal neuralgia, temporal lobe epilepsy, radiation necrosis, and carotid artery stenosis [48]. Complication rates may be skewed by the fact that many patients underwent craniotomies or radiotherapy prior to radiosurgery. Multiple groups have noted that postradiosurgical complications were more common in patients who previously received conventional radiotherapy [26, 46].

In summary, radiosurgery offers long-term tumor control in greater than 90% of patients with acromegaly, endocrine remission in approximately 50% of patients, and relatively low complication rates. Radiosurgery appears to have a shorter latency until functional response, and fewer side effects, than radiation therapy. In most major treatment centers, radiosurgery has replaced conventional external beam radiation therapy as the preferred adjunctive treatment in patients whose acromegaly is not controlled with surgery and GH-suppressive medications. Because of the latency in functional efficacy, risk of delayed recurrence, and small potential for adverse effects, close long-term followup is recommended in all patients who have received radiation-based treatments.  Table 2 summarizes the findings of various radiosurgical case series.

## 10. Postoperative Remission Criteria

While the goal treatment of acromegaly is the restoration of a normal pulsatile GH secretion pattern and normalization of serum IGF-1, the actual definition of hormonal is ever-changing. In the last decade, remission rates have been in evolution as hormone assays have become more sensitive [1, 29]. The Consensus on Criteria for Cure of Acromegaly 2010 defined biochemical cure to be (at 6 weeks postoperative followup visit) a random GH measurement of 2.5 *μ*g/liter or less, glucose-suppressed (nadir) GH less than 1.0 *μ*g/liter, and a normal sex- and age-matched IGF-1 level [12, 19, 29]. Krieger et al. found that a fasting morning GH level lower than 2 *μ*g/liter measured at postoperative day 1 was an important predictor of remission. This study concluded that although IGF-1 is an indicator of long-term disease control, a reliable assay for aiding in postoperative management of these patients is an early postoperative GH level.

## 11. Conclusions

Acromegaly is a life-threatening disease often requiring chronic interdisciplinary care by a skilled team of endocrinologists, neurosurgeons, ophthalmologists, and radiation oncologists, among others. The efficacy of transsphenoidal surgery to rapidly reduce GH hypersecretion in the treatment of acromegaly has been documented extensively. Although transsphenoidal surgery is often the preferred initial treatment for this disorder, patients nevertheless require appropriate long-term hormonal and imaging follow up to identify delayed recurrence and guide adjunctive radiation-based and medical treatment. In recent years, the advent of radiosurgery has provided another alternative in the overall treatment of patients with acromegaly. It is of utmost importance as a clinician to understand that the systemic effects of chronic GH excess are reversible if complete surgical remission and long-term normalization of GH levels can be achieved via multimodal surgical, medical, and radiosurgical therapy.

## Figures and Tables

**Table 1:** Preoperative factors correlating with surgical outcomes in acromegaly [7, 15].

Favorable outcomes	Unfavorable outcomes
Older age	Younger age
Preoperative GH level of <45 ng/mL	Preoperative GH level of >45 ng/mL
Noninvasive tumor	Invasive Tumor
Micoradenoma (≤10 mm)	Macroadenoma (>10 mm)
Densely granulated GH adenoma	Acidophil stem cell tumor

**Table 2:** Radiosurgery case series including greater than 50 acromegalic patients.

	Number of Patients	Type of radiosurgery	Mean marginal dose (Gy)	Acromegaly remission (%)	Mean followup (months)	Pituitary hormone deficiency (%)
Losa et al. 2008 [30]	83	GK	25 (goal)	60.2	69	8.5
Jagannathan et al. 2008 [26]	95	GK	22	53	57	34
Vik-Mo et al. 2007 [31]	61	GK	26.5	17	66	13
Voges et al.2006 [32]	64	LINAC	15.3*	37.5	81.9*	12.3*
Jezkova 2006 [33]	96	GK	32	44	53.7	27
Castinetti et al. 2005 [34]	82	GK	26.3	17	49.5	17
Kobayashi et al. 2005 [35]	67	GK	18.9	16.7	63.3	14.6
Jane et al. 2003 [36]	64	GK	15*	36	NR	28
Zhang et al. 2000 [37]	68	GK	31.3	96	>24	NR

*represents all adenomas in study not specific to growth hormone pituitary adenomas.

NR = value not recorded in study.
